# CXCL13/CXCR5 Axis Predicts Poor Prognosis and Promotes Progression Through PI3K/AKT/mTOR Pathway in Clear Cell Renal Cell Carcinoma

**DOI:** 10.3389/fonc.2018.00682

**Published:** 2019-01-22

**Authors:** Zaosong Zheng, Yuhong Cai, Haicheng Chen, Zhiliang Chen, Dingjun Zhu, Qiyu Zhong, Wenlian Xie

**Affiliations:** ^1^Department of Urology, Sun Yat-sen Memorial Hospital, Sun Yat-sen University, Guangzhou, China; ^2^Guangdong Provincial Key Laboratory of Malignant Tumor Epigenetics and Gene Regulation, Sun Yat- sen Memorial Hospital, Sun Yat-sen University, Guangzhou, China

**Keywords:** CXCL13, CXCR5, ccRCC, diagnosis, prognosis, progression

## Abstract

The chemokine ligands and their receptors play critical roles in cancer progression and patients outcomes. We found that CXCL13 was significantly upregulated in ccRCC tissues compared with normal tissues in both The Cancer Genome Atlas (TCGA) cohort and a validated cohort of 90 pairs ccRCC tissues. Statistical analysis showed that high CXCL13 expression related to advanced disease stage and poor prognosis in ccRCC. We also revealed that serum CXCL13 levels in ccRCC patients (*n* = 50) were significantly higher than in healthy controls (*n* = 40). Receiver operating characteristic (ROC) curve revealed that tissue and serum CXCL13 expression might be a diagnostic biomarker for ccRCC with an area under curve (AUC) of 0.809 and 0.704, respectively. CXCL13 was significantly associated with its receptor, CXCR5, in ccRCC tissues, and ccRCC patients in high CXCL13 high CXCR5 expression group have a worst prognosis. Functional and mechanistic study revealed that CXCL13 promoted the proliferation and migration of ccRCC cells by binding to CXCR5 and activated PI3K/AKT/mTOR signaling pathway. These results suggested that CXCL13/CXCR5 axis played a significant role in ccRCC and might be a therapeutic target and prognostic biomarker.

## Introduction

Renal cell carcinoma (RCC) is one of the most common cancers, representing ~3% of all adult malignancies (1). Clear cell RCC (ccRCC) is the most common histological type and accounts for about 70–75% of RCC (2). Despite the systemic therapy, 5-year survival rate for advanced ccRCC patients is less than 10%. Therefore, understanding the molecular mechanisms of ccRCC progression holds great promise in treatment of RCC.

Emerging evidence suggests that chemokine ligands and their corresponding receptors can directly or indirectly affect cell proliferation, migration, invasion, and metastasis to ultimately alter disease outcomes in cancer patients (3, 4). Identifying the functional roles of chemokine ligands may provide potential biomarkers and therapeutic targets for cancers patients (5, 6). Chemokine ligands can be divided into four groups based on their N-terminal cysteines, including CXC, C, CC, and CX3C chemokines (7). CXCL12/CXCR4 axis has been shown to play a significant role in prostate cancer and promoted cancer metastasis (8, 9). CCL2 targets vascular endothelial cells and affects tumor vascularization and metastasis through JAK2-STAT5 and MAPK pathway (10). Among the CXC chemokines, CXCL13, and its receptor, CXCR5, have been reported to be involved in the development of breast cancer, colon cancer, and lymphoma (11–13). To our knowledge, this is the first study to investigate the diagnostic and prognostic value of CXCL13 in ccRCC.

The PI3K/AKT/mTOR signaling pathway plays a significant role in cellular physiology and pathology. Previous studies demonstrated that PI3K/AKT/mTOR signaling pathway is one of the most frequently altered pathways in human cancer and dysregulation of this pathway contributes to cancer initiation and progression, including breast cancer, lung cancer, and renal carcinoma (14). The PI3K/AKT/mTOR pathway is highly activated in ccRCC and targeting this pathway, either alone or with other drugs, holds great potential in ccRCC treatment (15). However, the complex mechanisms of the activation of PI3K/AKT/mTOR pathway in ccRCC remains largely unknown.

In this study, we determined differentially expressed chemokine ligands between ccRCC tissues and normal tissues. We examined CXCL13 expression in both TCGA and clinical ccRCC cohort, and determined its diagnostic and prognostic value in ccRCC patients. We also explored the relationship between CXCL13 and CXCR5 expression, and whether CXCL13/CXCR5 axis could affect progression of ccRCC. Moreover, we tried to investigate the molecular mechanisms and highlight the significant role of PI3K/AKT/mTOR pathway in CXCL13/CXCR5 axis promoting ccRCC progression.

## Materials and Methods

### Computational Analysis of Chemokine Ligands in ccRCC Tissues

Gene Expression Profiling Interactive Analysis (GEPIA) was used to determine differentially expressed chemokine ligands between ccRCC tissues (*n* = 523) and normal tissues (*n* = 100). 534 ccRCC RNA-seq data and clinical information were downloaded from TCGA database. These data were used to analyze prognostic values of these chemokine ligands in ccRCC, and correlation between CXCL13 and CXCR5 expression in ccRCC tissues. Gene set enrichment analysis was used to determine associated pathways between different ccRCC groups.

### Cell Lines and Cell Culture

Human renal cell carcinoma cell lines ACHN, Caki-2, and 786-O and normal kidney cell line HK-2 were obtained from American Type Culture Collection (ATCC, Manassas, VA). ACHN and 786-O were cultured in RPMI 1640 medium (Gibco BRL, Gaithersburg, MD). Caki-2 was cultured in McCoy's 5a Modified medium. HK-2 was cultured in Dulbecco's Modified Eagle Medium/Nutrient Mixture F-12 (Gibco). All media were supplemented with 10% fetal bovine serum (FBS; Hyclone Technologies, Logan, UT). All cell lines were maintained in a humidified atmosphere with 5% CO_2_ at 37°C.

### Patients and Samples

90 pairs of ccRCC tissues and normal kidney tissues were collected with informed consent from patients who underwent radical or partial nephrectomy at Sun Yat-sen Memorial Hospital of Sun Yat-sen University. None of these patients received chemotherapy or radiotherapy. All these tissues stored in RNAlater at −80°C until RNA isolation. Serum sample were collected from 50 ccRCC patients and 40 healthy volunteers. Written informed consent was obtained from all participants.

### RNA Isolation, RNA Transfection, and Quantitative Real Time PCR

Total RNA from ccRCC cell lines or tissues were isolated using TRIzol reagent (Life Technologies, Carlsbad, CA) and reversed transcribed into cDNA using Primer Script RT Master (Takara Biotechnology, Dalian, China) according to manufacturer's instructions. Small interfering RNA (siRNA) targeting CXCR5 and non-targeting negative control (NC) were obtained from RiboBio Co (Guangzhou, China) and transfected into ccRCC cells using Lipofectamine RNAiMAX Reagent (Life Technologies) according to the manufacturer's instructions. The targeting sense sequence is 5′-GCAAGCTGAATGGCTCTCT-3′. Quantitative real time PCR was performed to examine gene expression with LightCycler 96 Real-time PCR instrument (Roche, Mannheim, Germany). β-actin was used as a normalizer and the 2^−Δ*CT*^ (ΔCT = CT value of β-actin—CT value of CXCL13 or CXCR5) method was used to compare gene expression levels. The sequences of primers were as follows: β-actin forward, 5′-ACTGGAACGGTGAAGGTGAC-3′. β-actin reverse, 5′-AGAGAAGTGGGGTGGCTTTT-3′. CXCL13 forward, 5′-GAGGCAAAGGAATCCATGTAGT-3′, reverse, 5′-TTCCCTGAGTATTCTATGAAGTCTG-3′. CXCR5 forward, 5′-CACGTTGCACCTTCTCCCAA-3′, reverse 5′-GGAATCCCGCCACATGGTAG-3′.

### Protein Extraction and Western Blotting

ccRCC cells were washed 3 times with ice-cold PBS and lysed in RIPA buffer (Thermo Fisher Scientific) containing protease and phosphatase inhibitor cocktail (Roche). Total proteins (30 μg) were separated on 10% SDS/PAGE gel and then transferred onto PVDF membranes (Merck Millipore, Burlington, MA). After blocking in TBST containing 5% skim milk, membranes were incubated with primary antibodies (Cell Signaling Technology, Danvers, MA) overnight at 4°C. The membranes were then washed 3 times with TBST, and incubated with secondary antibody for 1 h at room temperature. Protein expression was examined by a Molecular Imager system (Bio-Rad Laboratories) with an enhanced chemiluminescence kit (Merck Millipore).

### Enzyme-Linked Immunosorbent Assay

The serum CXCL13 expression of 50 ccRCC patients and 40 healthy volunteers were examined with a human CXCL13 enzyme-linked immunosorbent assay (ELISA) kit (Neobioscience Technology, China) according to the manufacturer's instructions. The results were examined with an ELISA reader Multiskan Mk3 (Thermo Fisher Scientific) at 450 nm.

### Cell Proliferation and Transwell Assays

CellTiter 96 Aqueous One Solution Cell Proliferation Assay kit (Promega, Madison, WI) was used to measure proliferative ability of ccRCC cells. 10^3^ cells were seeded into each well of 96-well plate and treated with 20 ul solution. Absorbance was measured at 490 nm using SpectraMax M5 (Molecular Devices, San Jose, CA). The cell proliferation was performed every 24 h and lasted 5 days. Transwell migration assays were performed using transwell chamber inserts (Corning, NY). 10^5^ cells were seeded into upper chambers in serum-free medium. After incubation for 24 h, cells in upper chambers were removed and cells migrating to lower chambers were fixed with methanol, stained with 1% crystal violet. Then the stained cells were counted in five randomly selected fields under a microscope.

### Statistical Analysis

Statistical analyses were performed using GraphPad Prism (version 6.0; GraphPad Software, Inc., La Jolla, CA) and SPSS (version 10.0; SPSS Inc., Chicago, IL) with *P* < 0.05 considered statistically significant. Student's *t*-test or one-way analysis of variance (ANOVA) was used to determine the significance of the differences between groups. Pearson's correlation was used to determine the correlation between CXCL13 and CXCR5. Kaplan-Meier method was used to determine the prognostic value of CXCL13 expression in ccRCC. Receiver operating characteristic (ROC) curve analysis was used to determine the diagnostic values of serum and tissues CXCL13 expression in ccRCC.

## Results

### Differentially Expressed Chemokine Ligands in ccRCC Tissues

**Figure 1:**
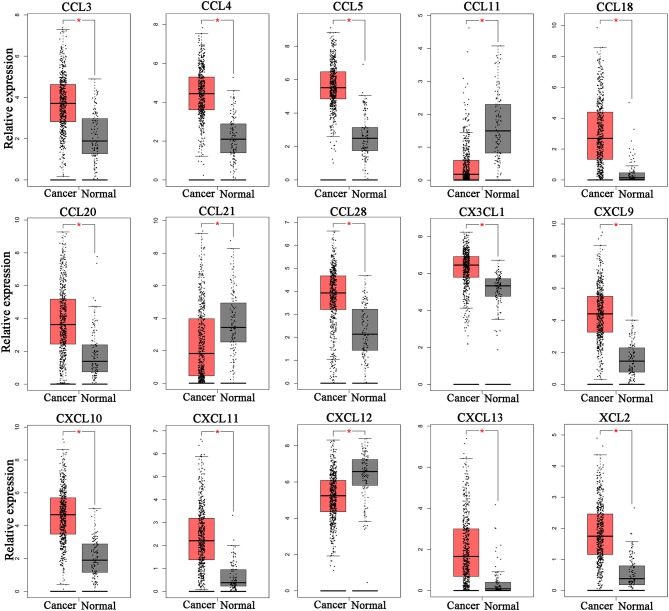
Differentially expressed chemokine ligands between ccRCC tissues and normal tissues. 12 chemokine ligands (CXCL9, CXCL10, CXCL11, CXCL13, CCL3, CCL4, CCL5, CCL18, CCL20, CCL28, CX3CL1, and XCL2) were upregulated in ccRCC tissues compared with normal kidney tissues. 3 chemokine ligands (CCL11, CCL21, and CXCL12) were downregulated in ccRCC tissues compared with normal kidney tissues. (Student's *t*-test, *P < 0.05).

To determine whether chemokine ligands are involved in the progression of ccRCC, we investigated expression of chemokine ligands in ccRCC tissues using GEPIA. The results revealed that 15 chemokine ligands (CXCL9, CXCL10, CXCL11, CXCL12, CXCL13, CCL3, CCL4, CCL5, CCL11, CCL18, CCL20, CCL21, CCL28, CX3CL1, and XCL2) were differentially expressed in ccRCC tissues (*n* = 523) compared with normal kidney tissues (*n* = 100) (Figure 1). These chemokine ligands might be involved in ccRCC progression and were selected for further analysis.

### Validation of CXCL13 Expression in ccRCC Tissues

**Figure 2:**
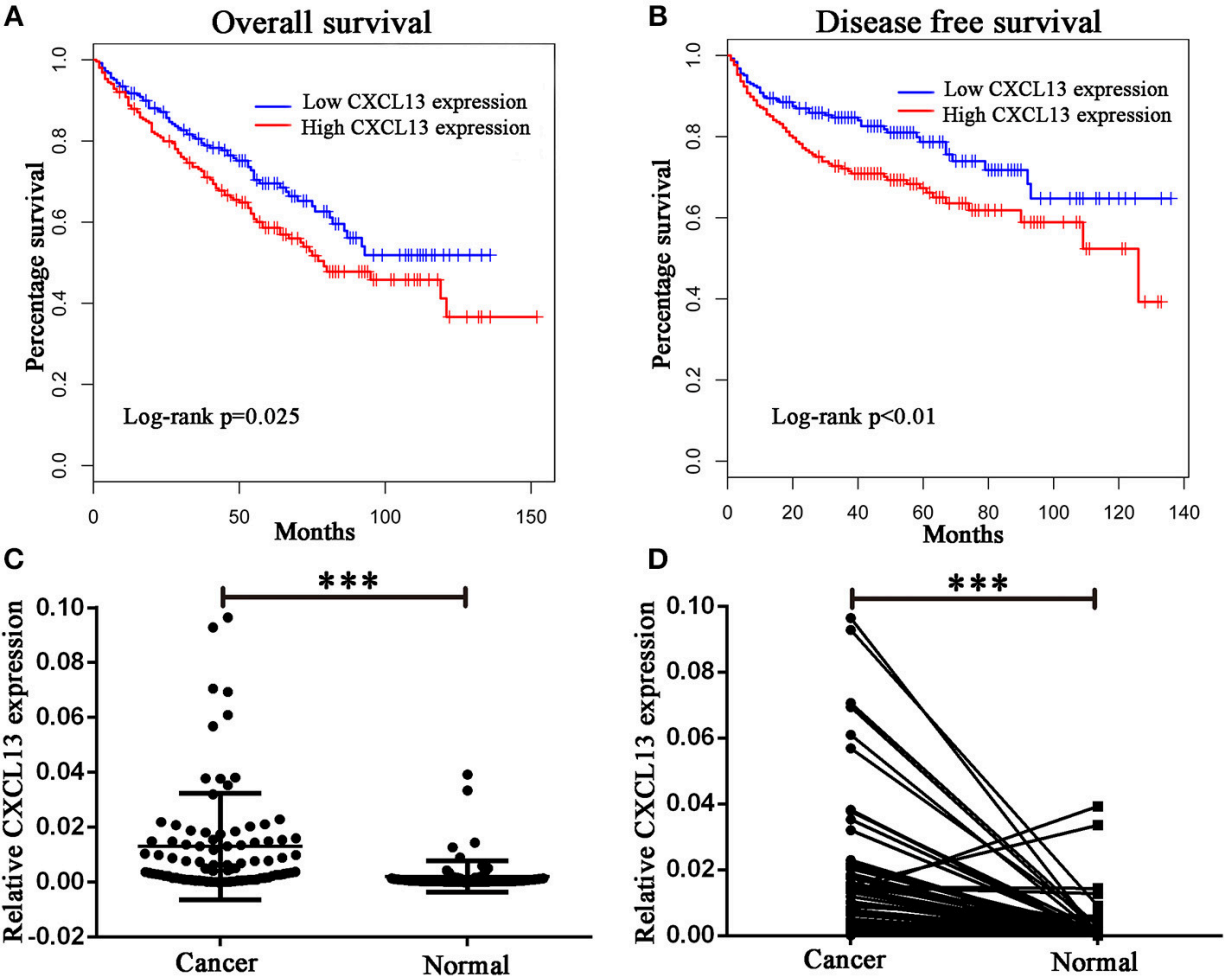
Validation of CXCL13 expression in ccRCC. **(A)** Kaplan-Meier analysis of overall survival in high-CXCL13-expression and low-CXCL13-expression ccRCC patients (Log-rank test, *P* = 0.025). **(B)** Kaplan-Meier analysis of disease free survival in high-CXCL13-expression and low-CXCL13-expression ccRCC patients (Log-rank test, *P* < 0.01). **(C)** and **(D)** Relative CXCL13 mRNA expression in 90 pairs of ccRCC tissues and normal tissues (Student's *t*-test, ***P < 0.001).

Then we analyzed the relationship between these chemokine ligands and the prognosis of ccRCC patients. We found ccRCC patients in high-CXCL13-expression group (*n* = 258) have a worse overall survival (Log rank *P* = 0.025) and disease free survival (Log rank *P* < 0.01) compared with ccRCC patients in low-CXCL13-expression group (*n* = 258) (Figures 2A,B). Therefore, we focused on the significant role of CXCL13 in ccRCC. We validated the result with a clinical cohort of 90 pairs of ccRCC tissues. RT-PCR revealed that CXCL13 was significantly upregulated in ccRCC tissues compared with normal tissues (*P* < 0.001) (Figures 2C,D).

### Correlation Between CXCL13 and Clinical Characteristics of ccRCC Patients

Then we analyzed the correlation between CXCL13 and clinical characteristics of ccRCC patients in TCGA cohort and our clinical cohort. In TCGA cohort, CXCL13 expression was significantly associated with tumor size (*P* = 0.022), T stage (*P* < 0.001), N stage (*P* = 0.004), and M stage (*P* < 0.001). No significant difference was found between CXCL13 expression and gender and age (Table 1). In our clinical cohort, in accordance with TCGA cohort, CXCL13 expression was significantly associated with T stage (*P* = 0.006), N stage (*P* = 0.017), and M stage (*P* = 0.044). No significant difference was found between CXCL13 expression and gender, age, and tumor size (Table 1).

**Table 1:** Correlation between CXCL13 expression and clinical characteristics of ccRCC Patients.

**Characteristics**	**Sun Yat-sen cohort**			**TCGA cohort**		
	**Number**	**Expression**	***P*-value**	**Number**	**Expression**	***P*-value**
Gender			0.216			0.146
Male	58	0.0160		345	5.184	
Female	32	0.0105		188	4.749	
Age			0.757			0.088
< 60	70	0.0167		246	4.765	
>60	20	0.0153		287	5.257	
Size (cm)			0.235			0.022
< 3	24	0.0098		458	5.044	
>3	66	0.0155		40	6.246	
NA				35	3.453	
T stage			0.006			< 0.001
T1-2	70	0.0110		342	4.406	
T3-4	20	0.0247		191	6.149	
N stage			0.017			0.004
N0	82	0.0125		239	5.057	
N1	8	0.0301		16	7.531	
NX				278	4.862	
M stage			0.044			< 0.001
M0	84	0.0129		421	4.873	
M1	6	0.0299		79	6.600	
MX				33	3.299	

### Diagnostic Value of CXCL13 Expression for ccRCC Patients

**Figure 3:**
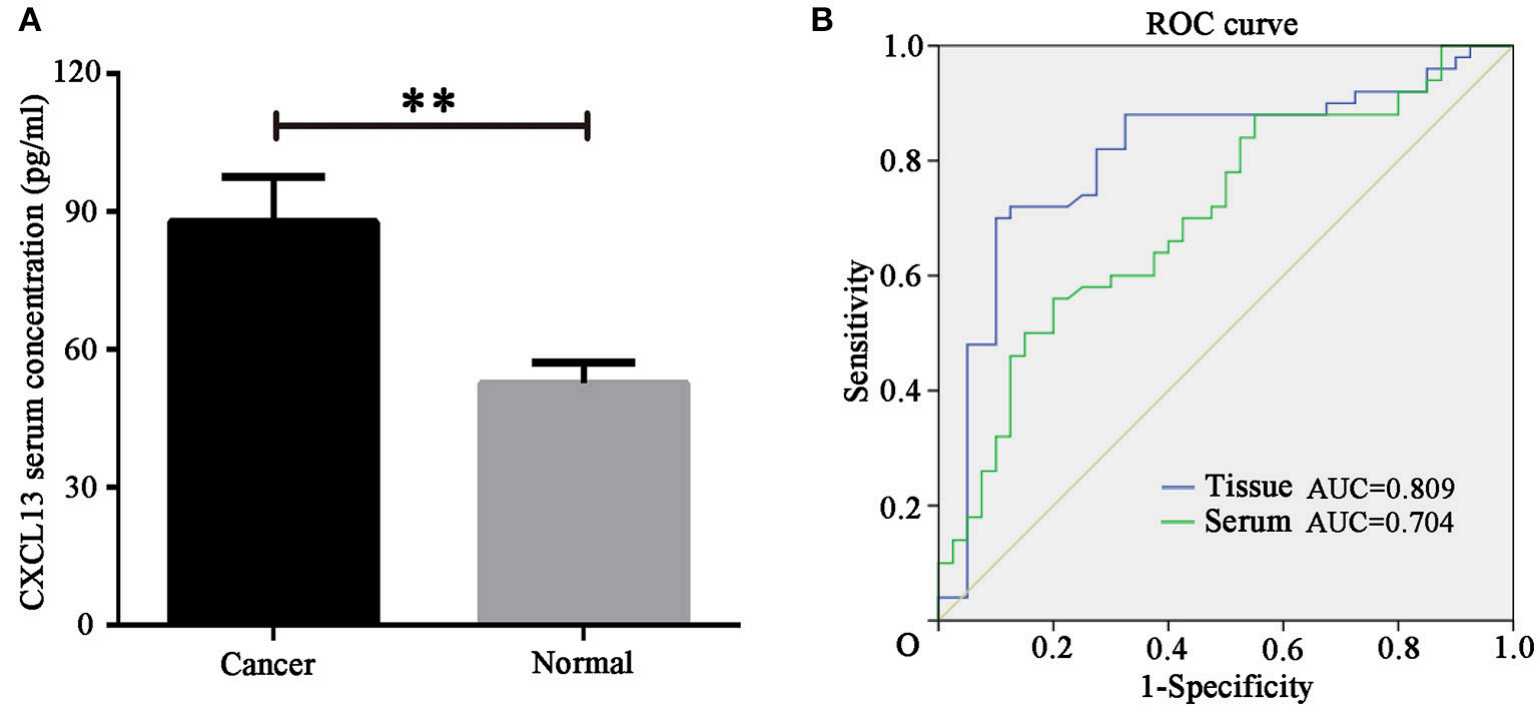
CXCL13 might be a diagnostic marker in ccRCC. **(A)** Serum CXCL13 level was determined with ELISA kit in ccRCC patients and healthy volunteers. Serum CXCL13 level was significantly higher in ccRCC patients than in healthy volunteers (Student's *t*-test, **P < 0.01). **(B)** ROC curve analysis of the diagnostic value of tissues and serum CXCL13 expression in ccRCC patients.

In order to identify the diagnostic value of CXCL13 in ccRCC, we determined serum CXCL13 expression in ccRCC patients (*n* = 50) and healthy volunteers (*n* = 40) using ELISA kit. The result showed serum CXCL13 expression was significantly higher in ccRCC patients than in healthy volunteers (*P* < 0.01) (Figure 3A). ROC curve revealed that tissue CXCL13 and serum CXCL13 expression might be a potential diagnostic biomarker for ccRCC with an AUC of 0.809 and 0.704, respectively (Figure 3B).

### Co-Expression of CXCL13 and CXCR5 in ccRCC

**Figure 4:**
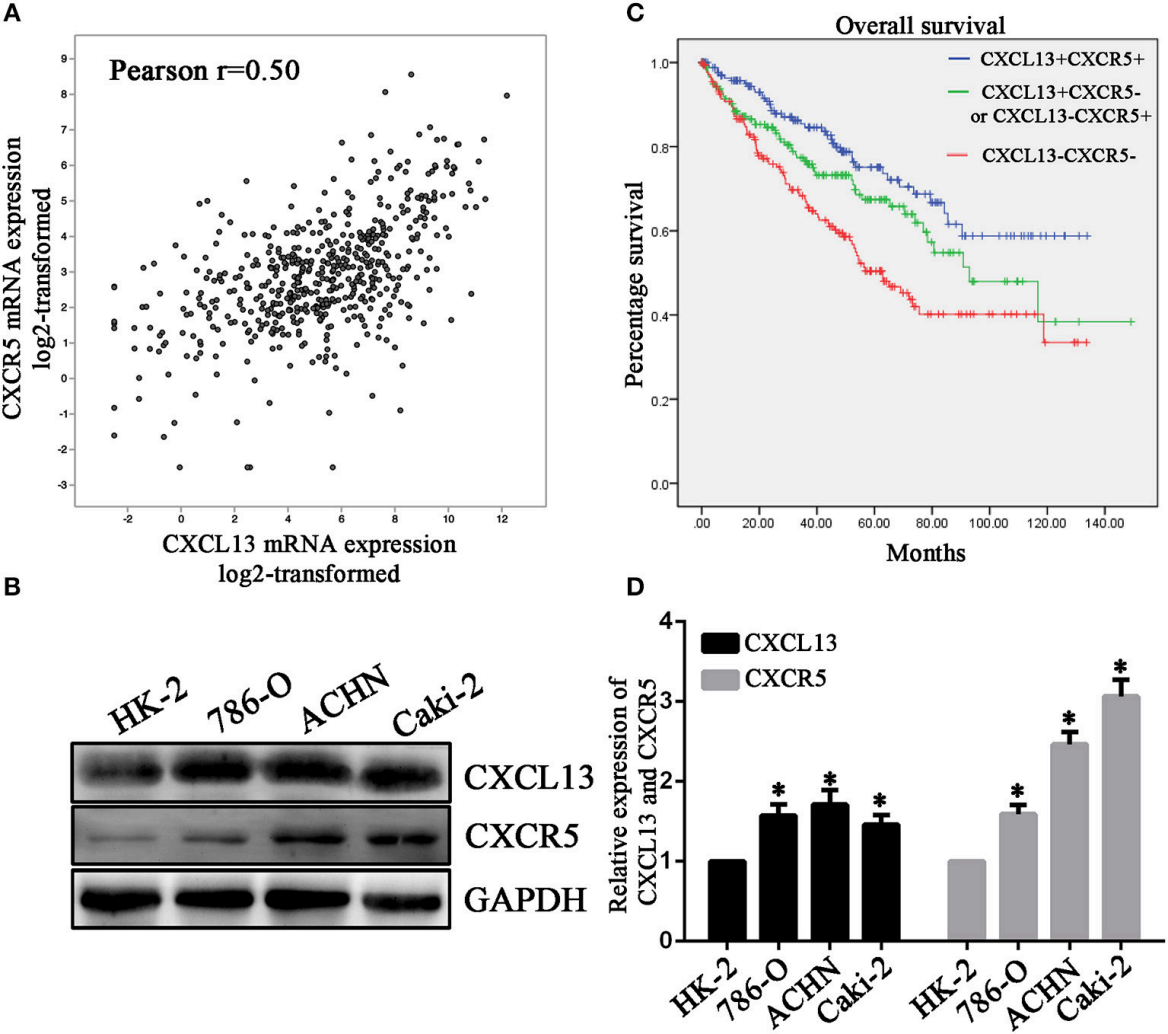
CXCL13/CXCR5 axis might be a potential prognostic marker in ccRCC. **(A)** Co-expression of CXCL13 and CXCR5 expression in ccRCC tissues (Pearson's correlation test, **P < 0.001). **(B)** Western blotting analysis of CXCL13 and CXCR5 protein level in cell lines. CXCL13 and CXCR5 expression were higher in ccRCC cells (ACHN, 786-O, and Caki-2) than in HK-2 (Student's *t*-test, *P < 0.05). **(C)** Kaplan-Meier analysis of overall survival in CXCL13^+^CXCR5^+^, CXCL13^+^CXCR5^−^/CXCL13^−^CXCR5^+^, and CXCL13^−^CXCR5^−^ ccRCC patients. **(D)** ccRCC patients in CXCL13^+^CXCR5^+^ group have a worst overall survival and ccRCC patients in CXCL13^−^CXCR5^−^ group have a best overall survival (Log rank test, *P* < 0.05).

Then we analyzed the association between CXCL13 and its receptor CXCR5 in ccRCC tissues. The results showed that CXCR5 expression was significantly correlated with CXCL13 expression in TCGA ccRCC tissues (*P* < 0.01) (Figure 4A). In addition, we examined CXCL13 and CXCR5 expression in ccRCC cell lines. The result revealed that both CXCL13 and CXCR5 expression were higher in ccRCC cells (ACHN, 786-O, and Caki-2) compared with normal kidney epithelial cell (HK-2) (Figure 4B).

### Upregulation of CXCL13/CXCR5 Axis Predicts Poor Prognosis in ccRCC Patients

To further evaluate the prognostic value of CXCL13/CXCR5 axis in ccRCC, ccRCC patients in TCGA database were divided into high CXCL13 high CXCR5 expression group (CXCL13^+^CXCR5^+^) (*n* = 178), low CXCL13 low CXCLR5 expression group (CXCL13^−^CXCR5^−^) (*n* = 177) and high CXCL13 low CXCR5 or low CXCL13 high CXCR5 group (CXCL13^+^CXCR5^−^/CXCL13^−^CXCR5^+^) (*n* = 178) according to the median value of CXCL13 and CXCR5 expression. Kaplan-Meier analysis revealed that ccRCC patients in CXCL13^+^CXCR5^+^ group have a worst overall survival and ccRCC patients in CXCL13^−^CXCR5^−^ group have a best overall survival (Log rank *P* < 0.05) (Figure 4C). These results suggested that CXCL13/CXCR5 axis might be a potential prognostic marker in ccRCC (Figure 4D).

### CXCL13/CXCR5 Axis Promoted ccRCC Proliferation and Migration

**Figure 5:**
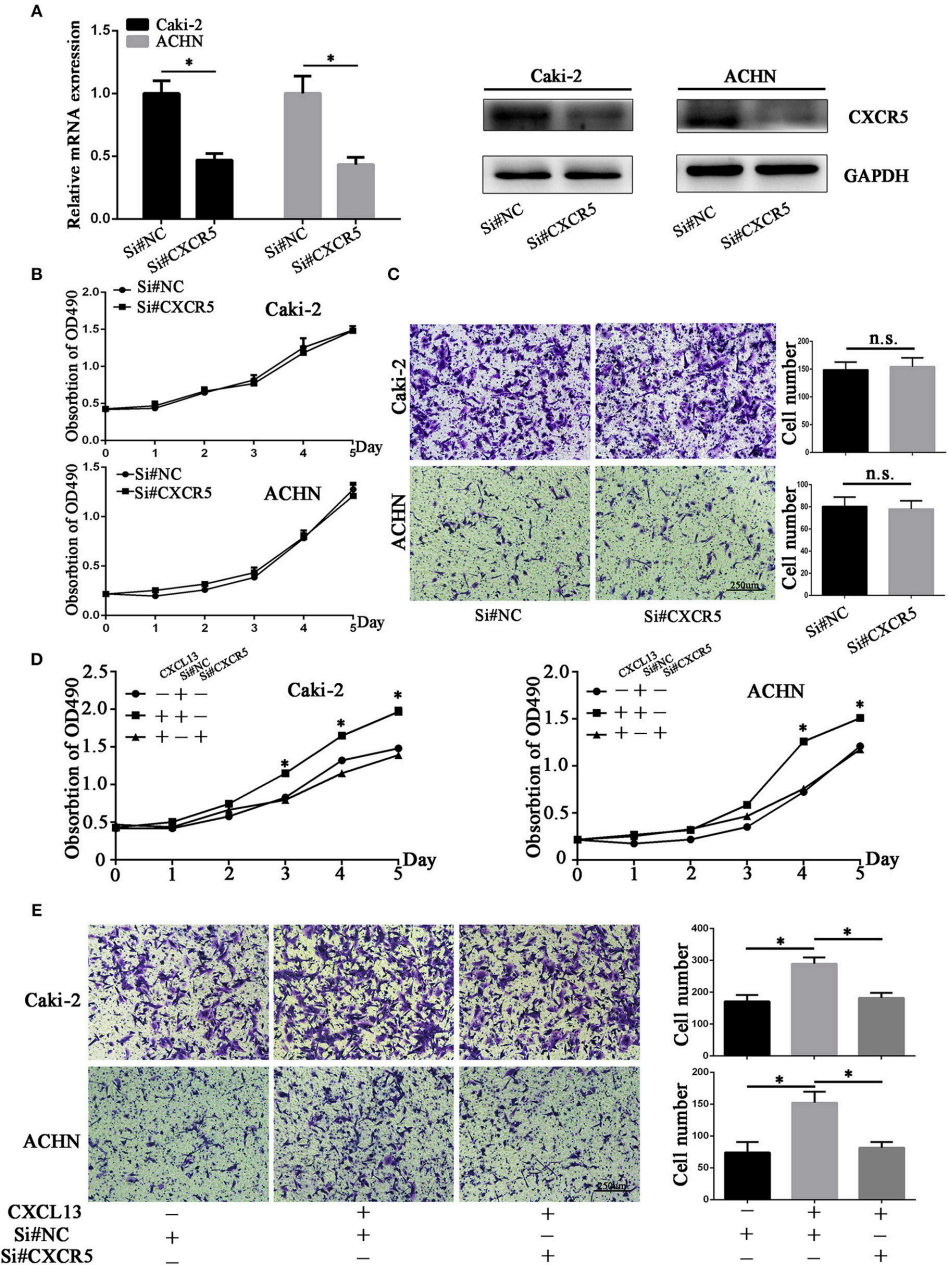
CXCL13/CXCR5 axis promoted ccRCC cells proliferation and migration. **(A)** CXCR5 mRNA and protein expression in ccRCC cells after transfection with si#NC and si#CXCR5(Student's *t*-test, *P < 0.05). **(B)** CXCR5 knockdown had no significant effect on proliferative ability in ccRCC cells. **(C)** CXCR5 knockdown had no significant effect on migratory ability in ccRCC cells. **(D)** CXCR5 knockdown inhibited the CXCL13-mediated cell proliferation in ccRCC cells as determined by CellTiter 96 Aqueous One Solution Cell Proliferation Assay (Student's *t*-test, *P < 0.05). **(E)** CXCR5 knockdown inhibited the CXCL13-mediated cell migration in ccRCC cells as determined by transwell migration assays (Student's *t*-test, *P < 0.05).

Then we aimed to explore the effect of CXCL13/CXCR5 axis on proliferation and migration of ccRCC cells. Small interfering RNA (siRNA) targeting CXCR5 was designed and CXCR5 expression was significantly downregulated by siRNA in ccRCC cells (Figures 5A,B). Cell proliferation and transwell assay revealed that CXCR5 knockdown had no significant effect on proliferative and migratory ability in ccRCC cells (Figure 5C). Then we stimulated ccRCC cells with CXCL13 (100 ng/ml) with or without CXCR5 knockdown at the same time. Cell proliferation assay showed that CXCL13 could dependently increase the proliferation of ccRCC cells, and CXCR5 knockdown could reduce the pro-proliferation effect of CXCL13 on ccRCC cells (Figure 5D). Transwell assays showed that CXCL13 could increase the migratory ability of ccRCC cells, and CXCR5 knockdown could reduce the pro-migration effect of CXCL13 on ccRCC cells (Figure 5E).

### CXCL13/CXCR5 Axis Regulated PI3K/AKT/mTOR Pathway in ccRCC Cells

**Figure 6:**
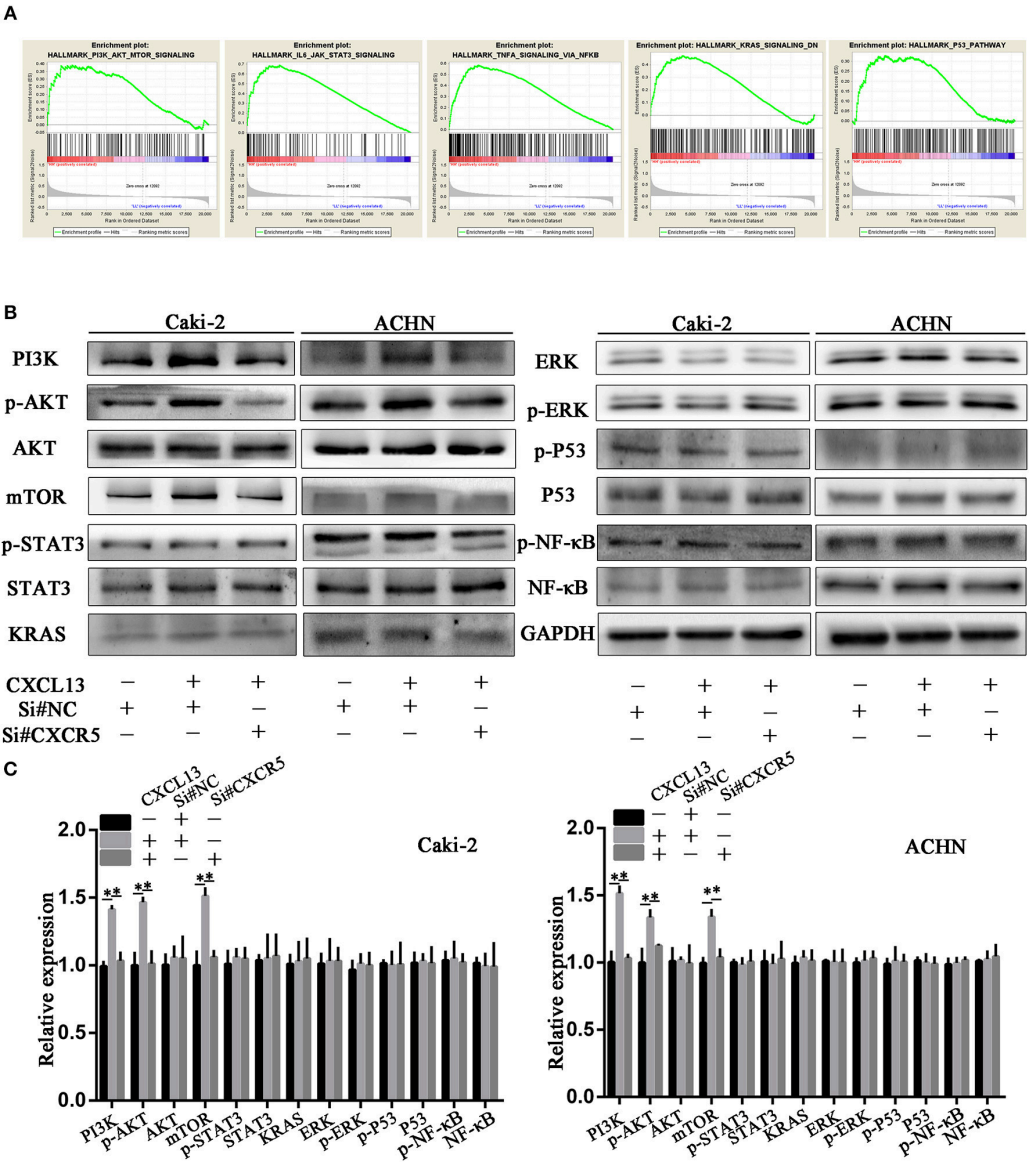
CXCL13/CXCR5 axis promoted ccRCC progression partly through PI3K/AKT/mTOR pathway. **(A)** GSEA was performed to identify associated pathways between CXCL13^+^CXCR5^+^ and CXCL13^−^CXCR5^−^ ccRCC tissues. PI3K/AKT/mTOR pathway, JAK/STAT3 pathway, TNF-α/NF-kB pathway, KRAS pathway, and P53 pathway were activated in CXCL13^+^CXCR5^+^ ccRCC tissues. **(B)** Western blotting analysis of these associated pathway in ccRCC cells. The expression of PI3K, p-AKT, and mTOR in ccRCC cells (Caki-2 and ACHN) were upregulated when incubated with CXCL13, and downregulated with CXCR5 knockdown. **(C)** Relative expression of associated pathway protein in ccRCC cells. GAPDH was used as normal control (Student's *t*-test, *P < 0.05).

In order to explore the underlying mechanism how CXCL13/CXCR5 axis promoted progression in ccRCC, we performed gene set enrichment analysis (GSEA). Gene profiles between CXCL13^+^CXCR5^+^ group (*n* = 178) and CXCL13^−^CXCR5^−^ group (*n* = 177) in TCGA database were compared. The result revealed that several cancer-related pathways, including PI3K/AKT/mTOR pathway, JAK/STAT3 pathway, TNF-α/NF-kB pathway, KRAS pathway, and P53 pathway, were activated in CXCL13^+^CXCR5^+^ group (Figure 6A). Then we performed western blotting to confirm whether CXCL13/CXCR5 axis promoted malignant behaviors of ccRCC cells through these pathways. The results showed that PI3K/AKT/mTOR pathway in ccRCC cells was activated when incubated with CXCL13, and suppressed when CXCR5 was downregulated (Figures 6B,C). No significant difference was found in JAK/STAT3 pathway, TNF-α/NF-kB pathway, KRAS pathway, and P53 pathway. These results indicated that CXCL13 could activate PI3K/AKT/mTOR pathway in ccRCC cells via its receptor CXCR5.

## Discussion

Chemokine ligands are small molecular weight (8–10 kDa) secreted proteins and are well known in regulating immunity, angiogenesis, and cell trafficking through binding their corresponding receptors (16–18). Increasing evidences suggested that chemokine ligands and their corresponding receptors are involved in cancer progression. CCL2 can recruit inflammatory monocytes to promote metastasis of breast cancer (5). CCL18 from tumor-associated macrophage facilitates cancer metastasis of ovarian cancer, breast cancer and pancreatic ductal adenocarcinoma (19–21). CXCL5 contributes to bladder cancer migration and invasion by binding to CXCR5, leading to upregulation of MMP2, and MMP9 (22). CXCL8-mediated signaling can promote survival of prostate cancer cell in hypoxic environments (23). However, few chemokine ligands have been well characterized in ccRCC.

In this study, we determined differentially expressed chemokine ligands in ccRCC tissues. Among these differentially expressed chemokine ligands, we found that CXCL13 was overexpressed in ccRCC tissues and cell lines, and significantly correlated with malignant stage and poor prognosis in ccRCC. CXCL13 has been reported to be involved in several cancer types, including prostate cancer, breast cancer, colorectal cancer, and lung cancer. In prostate cancer, CXCL13 mediates MMPs expression and actives protein secretion in a CXCR5-dependent way. Inhibition of CXCL13 or CXCR5 can impair the migratory and tumorigenic properties of prostate cancer cells (24). Serum level of CXCL13 is increased in metastatic breast cancer patients compared with normal donor and postoperative breast cancer patients (13). Here, we found that increased tissues and serum CXCL13 levels might be a diagnostic biomarker for ccRCC with AUC of 0.809 and 0.704, respectively. Our findings also demonstrated that ccRCC patients in CXCL13^+^CXCR5^+^ group had a worse overall survival and disease free survival compared with patients in CXCL13^−^CXCR5^−^ or CXCL13^−^CXCR5^+^/CXCL13^+^CXCR5^−^ groups, indicating that CXCL13/CXCR5 axis might be a potential prognostic biomarker for ccRCC.

Earlier research showed that chemokines targeted cancer cells and mediated cancer progression. For example, CXCL12/CXCR4 axis promoted stem-like properties, metastatic potential, and radiation resistance in cancer cells (25–27). CXCL8 enhanced resistance to anoikis in colorectal cancer cells, promoted castration-resistant through PI3K/AKT/mTOR pathway and regulating cyclin D1 in prostate cancer cells (28–30). CCL25/CCR9 signaling enhanced invasion and metastasis in breast cancer, melanoma, and ovarian cancer (31–33). In this study, we found that CXCL13 facilitated proliferation and migration of ccRCC cells and CXCR5 knockdown could attenuate the function of CXCL13, suggesting the significant role of CXCL13/CXCR5 axis on ccRCC progression.

CXCL13 could activate many signaling pathways in cancer cells through binding to its receptor CXCR5, a member of G protein coupled receptor (GPCR) superfamily. It is reported that CXCL13/CXCR5 axis promoted colon cancer growth and invasion via PI3K/AKT pathway (11), mediated prostate cancer cell proliferation through JNK and ERK signaling (34). In our study, we performed GSEA to screen associated pathways between CXCL13^+^CXCR5^+^ group and CXCL13^−^CXCR^−^ group and applied western blotting to validate these potential pathways. PI3K/AKT/mTOR pathway was activated when incubated with CXCL13, and suppressed when CXCR5 was downregulated, suggesting that CXCL13/CXCR5 promoted ccRCC progression partly through PI3K/AKT/mTOR pathway.

## Ethics Statement

This study was carried out in accordance with the recommen-dations of the Ethical Principles of Sun Yat-sen Memorial Hospital with written informed consent from all subjects. All subjects gave written informed consent in accordance with the Declaration of Helsinki. The protocol was approved by the Committee of Sun Yat-sen Memorial Hospital.

## Author Contributions

WX, ZZ, and YC conceived the study, designed experiments and wrote the paper. HC, ZC, DZ, and QZ performed experiments and analyzed data. All authors approved the manuscript.

### Conflict of Interest Statement

The authors declare that the research was conducted in the absence of any commercial or financial relationships that could be construed as a potential conflict of interest.

## Abbreviations

RCC: renal cell carcinoma
ccRCC: clear cell RCC
TCGA: The Cancer Genome Atlas
ROC: receiver operating characteristic
AUC: area under curve
GEPIA: Gene Expression Profiling Interactive Analysis
ELISA: enzyme-linked immunosorbent assay
SiRNA: small interfering RNA
GSEA: gene set enrichment analysis.
